# Exploring Barriers to Crisis Support: Considerations for the Design of Automated Digital Safety Planning Interventions

**DOI:** 10.1007/s41347-026-00678-4

**Published:** 2026-07-17

**Authors:** Arka Ghosh, Emily Tack, Sarah Popowski, Tanvi Lakhtakia, Theresa Nguyen, David C. Mohr, Kimberly A. Van Orden, Madhu Reddy, Jonah Meyerhoff

**Affiliations:** 1 Division of Clinical Informatics and Digital Transformation, University of California San Francisco, Suite 300, 10 Koret Way, CA San Francisco, USA 94134; 2 Center for Behavioral Intervention Technologies (CBITs), Department of Preventive Medicine, Feinberg School of Medicine, Northwestern University, Chicago, IL USA; 3 School of Psychology, Indiana University, Bloomington, IN USA; 4 Department of Psychology, University of Virginia, Charlottesville, VA USA; 5 Mental Health America, Alexandria, VA USA; 6 Department of Psychiatry, University of Rochester Medical Center, Rochester, NY USA; 7 Department of Informatics, University of California, Irvine, CA USA

**Keywords:** Suicidal thoughts and behaviors, Safety planning intervention, Text messaging

## Abstract

Suicidal ideation is common among young adults in the United States and represents an important mental health concern. However, formal mental health care utilization remains limited due to attitudinal and structural barriers. Digital mental health interventions (DMHIs) offer a promising approach to help-seeking, especially for young adults who prefer self-managing their symptoms. Text messaging-based safety planning interventions can be a scalable approach to seeking help that can mitigate many structural barriers for young adults. We conducted two online focus groups (*n* = 15 each) to understand what prevents young adults from seeking help for their suicidal thoughts and what might prevent them from engaging with a fully automated text messaging-based safety planning intervention. Participants reported barriers to help seeking both within and outside crisis states. Outside crisis states, the major barriers to help seeking include an unwillingness to burden others, inability to verbalize distress, fear of repercussions, stigma, and inconsistency with self-image. During crisis, participants reported difficulty following through with a safety plan. Despite a preference for self-management, participants expressed skepticism towards a fully automated intervention, citing privacy concerns, doubts about effectiveness, and a desire for human support. Understanding these barriers are critical for developing DMHIs that balance autonomy with perceived effectiveness and support.

**Supplementary Information:**

The online version contains supplementary material available at https://doi.org/10.1007/s41347-026-00678-4.

## Introduction

Suicidal ideation is common among young adults in the United States and represents an important mental health concern associated with distress, functional impairment, and elevated risk for future suicidal behavior (Han et al. 2018). Rates of suicidal ideation among young adults have increased over the past decade, particularly among individuals aged 18–25 years (Twenge et al. 2019). Despite high rates of mental health challenges, young adults have low rates of formal mental health treatment relative to their middle-age and older-adult counterparts (Blanco et al. 2008, Eisenberg et al. 2007, Lin and Parikh 1999). Many of the existing suicide prevention interventions are presently delivered via formal mental health care settings (e.g., outpatient and inpatient mental health clinics) (Hom et al. 2015, Larkin et al. 2024, O’Reilly et al. 2023). However, because many young adults face barriers to accessing these services, they are disadvantaged with regard to preventive care access (Gulliver et al. 2010, Rickwood et al. 2005, Rickwood et al. 2007). Many young adults experiencing suicidal thoughts do not seek formal treatment due to attitudinal barriers such as the belief that their problems are not severe enough to warrant additional help or that such treatment would not be helpful (Gulliver et al. 2010, Michelmore and Hindley 2012). Many also believe they should be able to manage suicidal thoughts on their own or prefer managing them without formal care (Gulliver et al. 2010, Luca et al. 2015, Reily et al. 2024). Other factors influencing whether a young person seeks help from others for their suicidal thoughts include structural barriers such as geographical convenience, resource availability, financial resources, and time (Hom et al. 2015, Ko et al. 2019, Mojtabai et al. 2011, Nada-Raja et al. 2003). Technological solutions can help mitigate these structural barriers and can satisfy young adults’ need for self-managing their suicidal thoughts.

Scalable digital mental health interventions (DMHIs) designed to support individuals experiencing suicidal thoughts have the potential to change how suicide prevention care is accessed (Beurs et al. 2015) and have demonstrated promise and acceptability (Melia et al. 2020, Torok et al. 2020, Witt et al. 2017). For example, Torok et al. reported that self-guided DMHIs directly targeting suicidal ideation are effective in reducing suicidal ideation immediately post-intervention (Torok et al. 2020). DMHIs focused on suicidal ideation and crisis self-management offer the ability to circumvent many of the help-seeking barriers young adults encounter when attempting to manage their suicidal thoughts: they are low cost, they can be used in private and on-demand. The majority of DMHIs targeting suicidal thoughts are apps or web-based interventions (Melia et al. 2020, Torok et al. 2020, Witt et al. 2017), which first require an individual to experience a desire and motivation to open a suicide prevention-specific app or website. In contrast, other technologies such as text messaging are “push” technologies, delivering a message directly to a user without the user needing to initiate the interaction. Text messaging has been shown to be a useful and acceptable medium for DMHIs that support management of suicidal thoughts (Comtois et al. 2019, Czyz et al. 2020, Czyz et al. 2021). Prior studies suggest that text messaging interventions can reinforce coping strategies, support use of safety plans, facilitate supportive contact following discharge from acute care settings, and promote engagement with suicide prevention resources among individuals at elevated suicide risk (Czyz et al. 2020, Czyz et al. 2021). Because text messaging is low burden, widely accessible, and integrated into daily communication patterns, it may represent a particularly feasible platform for delivering suicide prevention support to young adults. To date, text messaging-based interventions for suicidal thoughts have largely involved unidirectional messaging in which a system sends pre-populated messages to a user. However, text messaging is a bi-directional medium, which young people are using a great deal. Text messaging-based interventions for suicide prevention have been used to good effect (Czyz et al. 2020, Czyz et al. 2021). Delivering suicide prevention interventions via text messaging may increase intervention reach by removing the need to download purpose-built applications or visit dedicated intervention websites. One such intervention is the Safety Planning Intervention (SPI) (Stanley and Brown 2012), a brief, collaborative intervention that involves developing a personalized and prioritized list of coping strategies, sources of support, and resources that individuals can use during periods of heightened suicide risk. Safety planning has been associated with reductions in suicidal behavior, increased treatment engagement, and improved coping in clinical populations experiencing suicidal ideation and suicide risk (Nuij et al. 2021). As a result, SPI is widely recommended as a best-practice suicide prevention intervention in clinical settings (Kearns et al. 2025). However, evidence regarding its impact on suicide mortality specifically remains limited, and challenges related to implementation, engagement, and sustained use continue to be identified (Brown et al. 2024). As part of the present study, we aimed to understand how safety planning might be combined with interactive text messaging to ameliorate the many barriers to accessing suicide prevention care.

To effectively design a text messaging-based safety planning intervention that meets the needs of young adults who experience suicidal thoughts, we partnered with Mental Health America (MHA), one of the oldest and largest mental health patient advocacy organization in the United States. MHA is a nonprofit organization focused on mental health promotion, prevention, and support for individuals experiencing mental health concerns. MHA’s primary point of contact with their users is their national online website which hosts information about mental health symptoms, treatments, and prevalence. Young adults with lived experience of suicidal thoughts face low rates of help-seeking, but express interest in self-guided digital tools such as safety planning (Melvin et al. 2019, Methi et al. 2024, Rainbow et al. 2024). To better design these self-guided digital tools, specifically an automated text messaging-based safety planning intervention, we aimed to understand (1) the barriers that young adults face when reaching out for help when experiencing suicidal thoughts as well as (2) the anticipated barriers young adults who experience suicidal thoughts may face when engaging with a hypothetical automated text messaging-based safety planning intervention (Stanley and Brown 2012).

## Methods

### Participants

We enrolled 30 young adult participants (Table 1) into one of two (*n* = 15 each) asynchronous remote community (ARC) workshops (i.e., online asynchronous focus groups) (MacLeod et al. 2017, Maestre et al. 2018, Prabhakar and Colorado 2017). Our sample size was selected to achieve thematic saturation while ensuring sufficient diversity of perspectives (Guest et al. 2006, Hennink et al. 2017). Participants were recruited after visiting MHA’s website. MHA supported this study by providing feedback on the focus group questions, participating in regular meetings with the research team, and assisting with recruitment by hosting IRB-approved clickable advertisements. MHA was not engaged in the conduct of the research and did not have access to any identifiable participant information collected as part of the study.

Clickable recruitment advertisements were displayed to MHA site visitors who endorsed recent thoughts of self-harm or suicide on one of two self-screening questionnaires (the Patient Health Questionnaire or a non-suicidal self-injury screening tool), or after visiting an MHA webpage containing psychoeducational information about suicide prevention. Individuals who clicked on the advertisement were directed to a webpage containing a description of the study and a consent form for eligibility screening. Individuals who consented to screening were automatically directed to the eligibility screening questionnaires administered via REDCap (Harris et al. 2009, Harris et al. 2019). Eligible individuals were then provided with the opportunity to provide informed consent to participate in the study. Consenting individuals received instructions via email for enrolling in the online focus group platform. Participants were considered enrolled once they had successfully registered for the online focus group platform.

Participants were between the ages of 18 and 24 with sufficient English language proficiency to participate in all study activities, owned a smartphone, and experienced past two-week suicidal ideation as assessed as part of a research study screening via a modified version of the DSM-5 Cross-Cutting Symptom Inventory (American Psychiatric Association 2013) item assessing the recency of suicidal thoughts (i.e., “During the past two weeks, how much or how often have you been bothered by thoughts of actually hurting yourself or ending your life?”) and the Depressive Symptom Index - Suicidality Subscale (DSI-SS) (Stanley et al. 2021) to assess suicidal thought severity and disambiguation from self-harm-related thoughts. Exclusion criteria were active psychotic or manic symptoms in the last two weeks as assessed via the DSM-5 Cross-Cutting Symptom Inventory (American Psychiatric Association 2013), and potentially imminent suicide risk operationalized as the presence of recent intense desire to die as assessed via an item from the Beck Depression Inventory – II (2 or greater on BDI-II item 9) (Dozois et al. 1998). Mental health co-morbidity was also assessed using the DSM-5 Cross-Cutting Symptom Inventory and has been provided in Table S1 in the supplementary file. Across the two groups, 269 participants initiated the web-based screening process, 42 met eligibility criteria after completing screening, 38 completed the consent process, and 30 completed all enrollment procedures.

**Table 1:** Participant demographics

	Group 1	Group 2
Gender *n* (%)	-	-
Male	2 (13.3)	1 (6.7)
Female	9 (60)	13 (86.7)
Non-binary	4 (26.7)	1 (6.7)
Transgender n (%)	3 (20)	1 (6.7)
Race n (%)	-	-
Black/African American	2 (13.3)	1 (6.7)
American Indian/Alaska Native	1 (6.7)	0 (0)
Asian	2 (13.3)	3 (20)
Native Hawaiian/Pacific Islander	0 (0)	0 (0)
White	8 (53.3)	10 (66.7)
More than one race	1 (6.7)	1 (6.7)
Prefer not to answer	1 (6.7)	0 (0)
Hispanic/Latino n (%)	-	-
Yes	2 (13.3)	1 (6.7)
DSM-5 Cross-Cutting Symptoms M (SD) [0–4; frequency in last 2 wks.]		
Self-harm or suicidal thoughts	2.2 (0.9)	2.2 (1.0)
Depressive Symptom Index - Suicidality Subscale Total M (SD) [4 items each coded 0–3; range 0–12]	5.1 (0.96)	4.5 (2.0)

### Procedures

Eligible participants who provided informed consent were invited to participate in one of two online asynchronous focus groups. The first was held November through December 2022, the second between February and March of 2023. Online asynchronous focus groups were hosted using a research platform called FocusGroupIt. FocusGroupIt is an online service that facilitates qualitative user-centered design research with distributed groups by enabling researchers to create private forums where participants are able to view and respond to researcher posted questions as well as to one another, asynchronously. FocusGroupIt has previously been used as a platform in research with sensitive populations (Kruzan 2022, Reisner et al. 2018, Meyerhoff et al. 2022, Meyerhoff et al. 2025, Popowski et al. 2025). Participants registered for a FocusGroupIt account and were subsequently invited to the private online asynchronous focus group for this project. All participants were assigned anonymized usernames and were unable to view any personal details of other participants. In addition, participants agreed to a code of conduct that requested participants refrain from sharing any content or ideas shared by other participants outside of the focus group as well as refrain from attempting to establish connections with the other participants outside the study context. Once each group reached full enrollment, researcher-posted questions were released every 3 days. Each online asynchronous focus group comprised 8 total researcher-posted questions which broadly focused on the use of digital tools for symptom self-management, suicidal ideation, and safety planning. The questions were designed to address the overarching research question: What are the needs, preferences, and constraints of young adults experiencing suicide-related thoughts and behaviors with respect to self-management using digital tools? Participants were incentivized to reply to researcher-posted questions as well as one another to facilitate building a threaded conversation. Participants received $8 for responding to each researcher-posted prompt and an additional $2 for responding to at least one prompt posted by another participant, for a maximum of $10 per question. Online asynchronous focus groups were monitored by research staff daily for any posts that violated stated community guidelines, i.e., posts containing abuse, harassment, personal identifiable details, detailed descriptions of self-harm, suicide, or methods of self-harm or suicide. We reviewed the posts for identifying, harmful, or potentially imminent suicidal or homicidal ideation once a day on business days. Any posts violating these guidelines were removed. Participants were informed why the post was removed, and, if indicated, a risk assessment was conducted. Of note, only a single post was removed by the moderators due to concerns about invalidating language that conflicted with the posted community guidelines. No risk assessment was indicated as the offending post did not indicate potentially imminent suicidal or homicidal ideation. Transcripts of these online asynchronous focus groups were exported and analyzed. All procedures were approved by the senior author’s Institutional Review Board prior to participant involvement (STU00217191).

### Data Analysis

Data were analyzed using an inductive thematic analysis approach, a qualitative analytic method used to identify, organize, and interpret patterns of meaning within textual data without imposing predefined categories or theoretical frameworks (Braun and Clarke 2019, Braun and Clarke 2006). We selected an inductive approach because the primary aim of this study was exploratory, i.e. to understand participants’ needs, preferences, and constraints related to the use of digital tools for self-management of suicide-related thoughts and behaviors directly from participant narratives.

The analytic corpus consisted of transcripts from the online asynchronous focus groups, with each group containing 8 topic-based sessions. To focus the analysis on the primary study aims, we analyzed sessions most directly related to digital self-management and safety planning. Specifically, the corpus included: Online Asynchronous Focus Group 1 Sessions 3 (Safety Plans), 4 (Safety Plan and Text Messaging), and 5 (Automated Messages to Support Safety Planning), as well as Online Asynchronous Focus Group 2 Sessions 2 (When Suicidal Thoughts Arise), 3 (Safety Plans), 4 (Safety Plan and Text Messaging), and 5 (Automated Messages to Support Help Seeking). Details of all prompts are provided in Table S2.

The first author and senior author initially read through the entire corpus to familiarize themselves with the data and identify recurring concepts and patterns. The first author then conducted open coding on a subset of transcripts to generate an initial set of codes grounded in participant responses. Coding was conducted at the sentence or idea level within participant posts to preserve contextual meaning while allowing for detailed thematic analysis.

Using these initial codes, the first author developed a preliminary codebook, which was iteratively refined through discussion with the senior author. The first author subsequently applied the revised codebook to the full corpus. Throughout this process, the first and senior authors met on three occasions to review coded excerpts, discuss discrepancies or ambiguities in code application, refine code definitions, and revise the codebook as needed. These iterative discussions were conducted to improve consistency and ensure that the coding framework accurately reflected participant perspectives and the underlying data.

After coding was completed, the first author organized related codes into broader hierarchical and axial themes that captured recurring needs, preferences, and constraints discussed across participants. These themes were further refined through iterative review and discussion with the senior author until consensus was reached regarding thematic structure and interpretation. The analysis was considered complete once no additional substantive themes emerged from the data and the thematic framework adequately captured patterns observed across the corpus.

## Results

### Barriers to Young Adults Reaching Out for Help When Experiencing Suicidal Thoughts and Behaviors

Results from these analyses indicated two primary categories of barriers young adults face when considering reaching out for help and support when experiencing suicidal thoughts and behaviors. These include (1) barriers faced when young adults are outside of a crisis state and (2) barriers faced when they are in the midst of a crisis.

#### Barriers Outside of Crisis States

##### Not Wanting to Feel Like a Burden to Others

Young adults mentioned that asking for support from others often resulted in feeling like a burden, which was a key deterrent to reaching out for help. Participants reported being hesitant to reach out to close others who they knew cared for them because they did not want to burden their loved ones with their problems. As OFG1-P3 mentioned:

I know I have safe people in my life but I don’t want them to feel obligated to watch for signs from me or to have to take on me and my issues when I’m getting really bad.

OFG1-P3 highlights that by asking for support for their suicidal thoughts, they feel they are placing their loved ones in roles they did not choose and may not want to be in. This made it difficult for OFG1-P3 to ask for help.

In addition to the pervasive thought that by reaching out for support young adults could be inconveniencing, obligating, or otherwise burdening their loved ones, they were hesitant to reach out to others due to fears that loved ones would become hypervigilant. Specifically, they worried that whoever they reach out to might start to look out for warning signs of suicidal crises during routine interactions.

##### Negative Prior Experiences Reaching Out for Help and Support

Participants in our study also shared that negative past experiences seeking support served as a primary determinant of how willing they would be to reach out in the future. Negative experiences included invalidating statements following a request for support, being teased, and being involuntarily “outed” as someone who is suicidal. These negative experiences resulted in reluctance to reach out again and increased participants’ interest in self-managing their symptoms. OFG2-P5 stated:

I’ve often thought about reaching out and I still contemplate this thought. […] Most of the time I end up talking myself out of asking for help because anytime I make a mention of my mental health I’m shut down and told that I’m ‘making it up’ or ‘you just want attention’ or something more along the lines of ‘but you seem so happy’.

Conversely, participants who had reached out earlier and received support mentioned it was an important protective factor. As OFG2-P2 mentioned:

I made the decision to reach out to my mom and a few of my best friends because I felt horribly depressed and like I was drowning, and if I didn’t I was worried I was going to do something bad. I decided to tell those people because I know that they love and support me unconditionally.

Participants differed in how prior help-seeking experiences shaped future outreach behaviors. While some participants described invalidating or stigmatizing responses that discouraged future disclosure, others described supportive responses that increased their willingness to seek help during future crises.

##### Sense of Isolation

Another key barrier mentioned by young adult participants who experience suicide-related thoughts and behaviors relates to their sense of isolation and an inability for others to relate to their experiences. This barrier had two aspects to it. First, participants mentioned that they had difficulty verbally expressing their feelings and experiences as they relate to suicide-related thoughts and behaviors. Second, they believed that their social networks would not understand or relate to their experiences. As a result, participants assumed that their social networks would not be able to provide the necessary support even if they reached out to them. This inability to “feel heard” discouraged some participants from reaching out to friends and family. As OFG2-P13 stated:

I’ve decided not to mention it to my family because I don’t want to scare them and also they can’t really do anything to help. […] I’m not sure how to express it well honestly.

Young adult participants who experienced suicidal thoughts believed that their friends and family may not be able to help them. This perception prevented them from reaching out to their friends and family.

##### Cost Prohibitive Professional Help

High cost of accessing professional help was reported as a significant barrier. Young people who believed that they might benefit from professional mental health services (e.g., psychologist, psychiatrist, social worker, counselor, or other licensed therapist), did not seek it out when it was not adequately covered by their health insurance plan. In addition, several participants who accessed mental health professionals through their workplace or school (e.g., employee assistance programs, college counseling centers) were forced to discontinue services upon leaving their organization. OFG2-P14 said:

I had sought professional help but could not anymore because it was too expensive (with health insurance and everything). Before, I was able to see my therapist for free through my college, but now, with my health insurance, therapists would either not be covered or it would cost around $150 per session, which is what I cannot afford. So currently, I decided not to reach out to professional mental health providers.

OFG2-P14’s statement highlights that although young adults may derive benefit from professional services, cost is a serious consideration and when insurance coverage is insufficient individuals exit the market for services despite a persistent need.

##### Fear of Repercussions for Disclosing Suicidal Thoughts and Behaviors to Professionals

Across young adults in our study, including those who endorsed accessing support from mental health professionals, fear of negative repercussions or carceral responses to disclosure of suicide-related thoughts and behaviors to mental health professionals was another significant barrier to professional mental health help seeking. Young people expressed concerns about confidentiality when talking with mental health professionals, specifically noting fear that they might be reported to authorities and/or involuntarily hospitalized. Participants noted that mandatory reporting laws and young people's understanding of these laws and how they are implemented in cases of suicide-related risk served as a structural factor that discouraged disclosure. As OFG2-P13 mentioned:

I have recently begun working with a counselor through the program offered at my university, but I am very hesitant to be completely honest with her about my suicidal thoughts because I don’t know what she’ll do if I tell her. I’m attending school away from my home state, and I’m not sure what the reporting laws are.

Some participants who sought professional services refrained from disclosing suicidal thoughts due to uncertainty regarding specific regulations, policies, or practices guiding a clinician’s possible responses to disclosure of suicidal ideation.

##### Cultural Stigma

Young adult participants cited culturally bound mental health stigma as a barrier to seeking help since reaching out for help can result in harsh judgement, unfair treatment, ostracization, or other negative social consequences. This is especially true in certain cultural contexts. Young people in our study cited cultural beliefs around mental health-related issues as a core barrier to reaching out for help and support. Young adult participants shared that the people they love and trust and might consider reaching out to for support could have cultural beliefs around mental health that preclude them from acknowledging participants’ experiences of mental health symptoms and creating a validating environment conducive to disclosing suicide-related thoughts and behaviors. As OFG2-P14 stated:

Within my culture, I have a sense of immense vulnerability if I was to address my suicidal thoughts and feelings to people that I am close with. I know that some people will not judge me, however, even if I put their name down in my safety plan, I feel scared to reach out to them.

As mentioned by OFG2-P14’s statement, if it is not culturally acceptable to speak openly about mental health concerns, young adults tend to avoid seeking help.

##### Outreach and Self-image

Importantly, participants highlighted that reaching out for help would be inconsistent with how they think others see them. This inconsistency between their perceived self-image and the act of reaching out for support resulted in participants sharing strong resistance to the idea of reaching out to others for support until they have hit an intractable low point in their mood or ability to survive a suicidal crisis. OFG2-P12 mentioned:

I have always presented myself as a very strong and independent person, so to admit my struggles and to put myself in this position of vulnerability has been difficult.

Participants found it difficult to reach out for help as it conflicted with their perceived self-image.

#### Barriers During Times of Crisis

##### Difficulty Contacting Others

Young adult participants noted that when they were in a crisis state, they have difficulty actually contacting others they planned to reach out to for support when they were not in a crisis state. Specifically, the act of communicating that support is needed while in the midst of a crisis state can be overwhelming and exceptionally difficult. As a result, the crisis state itself becomes a significant barrier to outreach. However, participants spontaneously generated potential solutions to support overcoming this barrier. For example, OFG1-P1 said:

It’s so easy to think of a plan when you are in a happy state, but it’s a whole other level to use that when you are struggling. I think I would be more likely to use it if my “crisis people” knew what it meant when I reached out saying I’m using my safety plan- that way I wouldn’t have the added stress of explaining.

As highlighted by OFG1-P1’s response, a critical barrier to reaching out for help during times of crisis is whether or not an individual has social contacts who are already familiar with their experiences of suicidal thoughts. The crisis state itself becomes a difficult time to disclose suicidal thoughts to a trusted other.

### Barriers to Young Adults Who Experience Suicide-Related Thoughts and Behaviors Engaging with an Automated Digital Safety Planning Intervention

Data from our study show that perceived barriers faced by young adults who experience suicidal thoughts when considering an automated text messaging-based safety planning service included barriers related to (1) privacy, (2) effectiveness, and (3) the desire for human support.

#### Privacy

When participants considered receiving support from an automated text messaging-based intervention, they reported concerns about privacy. Participants appreciated the affordances that a text messaging-based intervention might provide such as the ability to engage with it privately and easy access compared to a phone conversation with a human crisis counselor at a helpline. However, participants highlighted concerns that text messages about suicide could be read by third parties with access to participants’ physical smartphone and the subsequent accidental disclosure of suicidal thoughts that would result. As OFG1-P10 mentioned:

If someone I didn’t know saw that on my phone, I would be worried about getting judged from the stigma that still very much surrounds mental illness.

Furthermore, participants expressed concern whether it is possible for a text messaging-based service to be fully anonymous as their phone number would still be linked to their messages and could potentially be traced back to them. As OFG2-P9 said:

I feel like my concern is not as high that someone would see it, but there is a fear of being traced and how it is not completely anonymous because the phone number is linked to you.

Participants expressed different types of privacy concerns related to text messaging interventions, with some emphasizing concerns about accidental disclosure through others viewing messages on their phones, while others were more concerned about anonymity and digital traceability. While participants indicated that they might be more willing to share their experiences and perspectives with a non-judgmental automated program compared to a crisis helpline worker, they would need assurance that the automated program maintained their anonymity. Participants indicated such systems should be explicit to the user about any limits to confidentiality.

#### Concerns About Effectiveness

Young people who had previously created a safety plan and did not find it useful expressed skepticism about the utility of creating another safety plan. Participants mentioned that safety plans that fail to prioritize their preferences and abilities were not likely to be useful in moments of need. Participants emphasized the need for creating safety plans using a collaborative process such as a therapist who plays a supportive role eliciting responses from a patient rather than playing an active role in creating a plan for a patient. As OFG2-P6 stated:

The truth of the matter is that I know myself best, and I think safety plans work best when they are mainly constructed by the person in need of the plan with support from the therapist and not the other way around.

Participants who had previous experience with a supportive therapist who facilitated the collaborative development of a highly personalized safety plan shared that the collaborative process made them more willing to use their safety plans and create additional safety plans in the future. As OFG2-P16 stated:

We sat and worked on it together, we went back and forth on things, they helped me to see what other people in my life can be a part of my safety plan.

However, when young adults imagined creating a safety plan with the help of an automated system, some of them were skeptical of the idea and felt that an automated system would not be able to help create a personalized, clear, and meaningful safety plan. While they appreciated the convenience a text messaging-based platform would provide, they doubted that a meaningful safety plan could be created without the involvement of a trained professional who has some insight into the individual’s problems. As OFG1-P4 stated:

*I like the idea how much more accessible it would be for phone users since that’s what most people spend their time on. But I feel like*,* it would work better with help of a therapist just to have someone with you in the process for questions or another perspective.*

Participants varied in their perceptions of the effectiveness of safety planning intervention. While some participants expressed skepticism based on prior experiences with generic safety plans, others described collaboratively developed plans as highly valuable and supportive. Overall, participants were open to the idea of using text-messaging to create a safety plan but were skeptical that a fully automated program could be sufficiently personalized or collaborative.

#### Absence of Human Support

When young adults imagined the experience of getting support from an automated digital safety planning tool, they shared that the automated nature of the program could serve as a barrier to engagement. The act of working collaboratively with another human may serve as a motivator for engagement because another person is dedicating time and effort to them, specifically. Young people cautioned that the absence of human involvement in a suicide prevention program and the nature of text messaging could serve to make the experience of creating a safety plan de-personalized which could in turn become a barrier to use. As OFG2-P3 mentioned:

However, one potential drawback of a safety planning tool delivered over text messaging is that it is depersonalized, and when experiencing feelings of loneliness/isolation alongside suicidal thoughts, it is especially beneficial to have social support mechanisms in place.

Some participants anticipated that a lack of involvement of a human in the process might worsen their feelings of isolation and might make them more likely to ignore the messages. In general, participants desired some form of human support while creating a safety plan.

Overall, participants expressed mixed views regarding automation. Some participants appreciated the potential privacy and accessibility benefits of an automated text messaging intervention, whereas others worried that reduced human involvement could increase feelings of isolation or make the intervention feel impersonal.

## Discussion

We have presented the results of two online asynchronous focus groups conducted to understand the barriers faced by young adults while seeking help for suicidal thoughts and to elicit their preferences for a fully automated text messaging-based safety planning intervention. Young adult participants with a history of suicidal thoughts highlighted several structural and attitudinal barriers that prevent them from obtaining support for their suicidal thoughts. Young adult participants reported that they struggle to reach out for support, experience social isolation, and encounter unsupportive responses to disclosures of suicidal thoughts. Moreover, participants in our study reported that cultural stigma, and high cost makef it difficult to find and access both professional and informal support. In addition, young people in our focus groups highlighted critical barriers related to privacy, effectiveness, and human support that might prevent them from using a digital suicide prevention intervention.

A sense of social isolation and a perception that one would become a burden to others by reaching out for help are major factors that prevent young adult participants in our study from seeking support for their suicidal thoughts. The Interpersonal Psychological Theory of Suicide (IPTS) highlights that social disconnection and perceived burdensomeness drive suicidal thoughts and behaviors (Orden et al. 2010), which raises the possibility that social disconnection and perceived burdensomeness may also play an indirect role in suicide risk by impacting help-seeking and coping behaviors. Participants in our study experienced social isolation and noted that social isolation served as both a driver of suicidal thoughts as well as a maintenance factor that made it more difficult to reach out to others during a crisis. Additionally, participants in our study viewed reaching out to others for support as potentially burdening their social networks, motivating the desire to use self-management tools. Perceptions of burdening others has been studied in the context of decision-making for end-of-life decisions (including desire for hastened death): findings indicate that the association between perceived burden and the desire for death is complex (Gudat et al. 2019). While some people reported that perceived burdensomeness increased their desire for death (or hastened death), others believed that their death would be a greater burden, which strengthened their will to live. Our research highlights the relevance of this dynamic (between perceived burdensomeness and a desire for death) for help-seeking behaviors in young people and highlights the need for additional study. Fears of burdening one’s social network and feeling like one does not belong can both foster a desire to use self-management tools to reduce the perception that one is burdening others, while also making it more difficult to derive benefit from those tools because that same perception of burdensomeness that motivates a desire for using self-management tools also motivates a desire for death.

Previous stigmatizing encounters also prevented young people in our study from reaching out for support. Participants in our study shared the difficulty of communicating about suicidal thoughts with individuals in their network who are perceived to hold negative or moralistic views about mental health symptoms, leading these young people to experience a smaller social support network. Cultural stigma led young people in our study who had lived experience of suicidal thoughts to repeatedly encounter unhelpful comments from others in their network who may view themselves as attempting to be supportive, leading to worsening symptoms and deepening social isolation. Stigma can be especially limiting for young adults from cultures where social networks are not sensitized about mental health problems which can result in ostracization (Grover et al. 2020).

Young adult participants had mixed experiences with creating a safety plan. Young adults who had a supportive and collaborative therapist found the experience and the resulting safety plan useful, while those who did not have a supportive therapist ended up with a safety plan that did not help them in times of need. Young adults in our study mentioned multiple barriers that prevented them from seeking help from humans in their network and they expressed a desire for self-reliance via digital mental health tools. However, while imagining support from an automated digital intervention, they felt that an automated intervention would not be able to help them create a personalized safety plan, and the lack of human support might exacerbate feelings of isolation and loneliness. There is a tension in the pursuit of increasing access to evidence-based care between automated digital suicide prevention interventions and digital suicide prevention interventions that involve a trained counselor who may be able to help personalize safety plans. This finding is echoed in studies of other young people’s attitudes toward digital mental health interventions. While there is low appetite for formal mental health treatment, automated digital mental health interventions, by virtue of excluding human support, may be ideal for some, and insufficient for others (Byron 2019, Meyerhoff et al. 2025, Reyes-Portillo et al. 2022, Poll et al. 2023). Understanding the nature of the relationship between self-guided digital suicide prevention intervention use and subjective feelings of loneliness and isolation is necessary in future work to better characterize the potential risks of self-guided suicide-prevention technologies that aim to increase access to evidence-based care. While involving young people with lived experience of suicidal thoughts directly in the design process mitigates some of the risk that resultant digital interventions will worsen symptoms, considering alternative models of care delivery may also be necessary (e.g., hybrid, or limited human-supported digital suicide prevention interventions).

The tension between desiring self-guided tools and skepticism about their effectiveness raises important design considerations for automated digital suicide prevention interventions. Currently, individuals in the US can access suicide prevention support through healthcare providers, crisis helplines, emergency services, loved ones, and self-management strategies; however, many people experiencing suicidal thoughts face significant barriers to engaging with these resources. The proposed text messaging-based safety planning intervention aims to support active coping with suicidal thoughts. One possibility might be to include a blended approach to managing imminent suicide risk within self-guided digital suicide prevention interventions may increase acceptability. For example, combining self-guided resources (e.g., on-demand safety plan review, step-by-step guides for involving trusted others) with referrals to human-supported crisis services like the National Suicide and Crisis Lifeline (9-8-8), Crisis Text Line, Trevor Lifeline, and Trans Lifeline. Digital self-guided interventions often contain a “one-click” connection to these services, 9-1-1, or emergency services. This approach enables these human-supported crisis resources to become extensions of self-guided interventions in moments when self-guided tools are not the optimal or desired option for an individual. However, given that there are potential harms when emergency services are involved inappropriately (Denneson 2024, Ward-Ciesielski and Rizvi 2021) the digital intervention must preemptively provide psychoeducation on the differences between emergency services and crisis helplines, enabling users to determine the most appropriate resource to use and when.

Our results also highlight that privacy is of utmost importance for young adults. Without transparent information about confidentiality, and the limits thereof, young people were especially reticent to seek help and support. This includes disclosing how and when clinicians or systems determine when to break confidentiality. To engage with a digital intervention, young adults need to feel that their privacy will be respected, and that the data collected during routine use of the intervention will not be used in a way that causes harm or potential harm to the individual by being shared with others without their consent or used in non-consensual carceral responses. To this end, simple, clear, and transparent privacy practices are needed and should be conveyed to users of an intervention at multiple points before and during the delivery of an intervention. Young adults need to feel assured that they, and not the service providers, are in control of their data. The service should allow users different degrees of data sharing and should allow them to easily understand how their data is being used and maintain the option of deleting their data. Additionally, young adults were concerned about others reading notifications from the program on their phone. This concern about accidental disclosure is important to consider as part of the design of an intervention. For example, it is possible to hide notifications on lock screen and add a PIN for sensitive apps. That might prevent someone from accidentally seeing notifications.

Digital interventions, broadly, and text messaging interventions, specifically, may hold promise for supporting individuals experiencing suicidal thoughts. Text messaging is a familiar and frequently used communication medium among young adults (Inc 2014, Smith 2015) and may offer a way of engaging with suicide prevention content that addresses some common barriers to care, including privacy, convenience, and accessibility. Prior work suggests that text messaging-based suicide prevention interventions are generally acceptable to users (Comtois et al. 2019, Czyz et al. 2020, Czyz et al. 2021, Berrouiguet et al. 2018, Pisani et al. 2019, Thiha et al. 2016), although additional work is needed to establish their efficacy and effectiveness for suicide-related outcomes. Interventions addressing young adults’ needs and preferences for suicide prevention must be co-designed (Kruzan et al. 2021).

In the present project we conducted two online asynchronous focus groups to elicit end-user preferences for an automated text messaging-based safety planning intervention. Digital suicide prevention interventions such as automated text messaging-based safety planning have the potential to address some of the key structural and attitudinal barriers. They can be low-cost or free, can be used to self-manage symptoms by being used in the moment, and can be used privately.

### Limitations

This study is not without limitations. First, while we incorporated the perspectives of 30 young adults with lived experience of suicidal thoughts, these were participants who were all interested in digital self-management approaches for suicidal thoughts and completed an eligibility screening and enrollment processes including an informed consent procedure. Their perspectives may not be representative of the broader young adult population with lived experience of suicidal thoughts. Second, our results are representative of young adult MHA users who experienced suicidal thoughts and might not be generalizable to all young adults who experience suicidal thoughts. Third, while we aimed to surface ideas, opinions, needs around suicide prevention technology via our online asynchronous focus groups, researcher-posted questions within each online asynchronous focus group generally focused on aspects of a text messaging-based intervention, which may have limited the responses we elicited. Finally, the ideas and opinions expressed in this study are extracted from stated, as opposed to demonstrated, preferences about a hypothetical digital suicide prevention and past experiences with suicide-related thoughts and behaviors and may not be representative of how young people with suicidal thoughts make use of or want to interact with a digital suicide prevention intervention.

### Conclusions

Young adults face multiple structural and attitudinal barriers to accessing help for suicidal thoughts both prior to and during crisis situations. While imagining support from an automated text messaging-based intervention, young adults had mixed reactions. They recognized the potential benefits of a fully automated intervention including accessibility and ease of use but expressed concerns about the privacy of their data and the effectiveness of such an intervention. Further, they reported that a lack of human support in moments of crisis might exacerbate young adults’ feelings of isolation. Future research should address these concerns and evaluate whether automated text messaging-based interventions can effectively improve suicide prevention outcomes while remaining acceptable and engaging to young adults.

## Supplementary Information

Below is the link to the electronic supplementary material.

Supplementary Material 1

## Data Availability

The data that support the findings of this study are available from the corresponding author upon reasonable request.

## Declarations

### Author area of Interest

Digital mental health, suicide prevention research.
