# Supplementary material for: Exploring Barriers to Crisis Support: Considerations for the Design of Automated Digital Safety Planning Interventions
Source: J Technol Behav Sci. 2026 Jul 17;11(3):592–604. doi: 10.1007/s41347-026-00678-4 (PMC13470603; doi:10.1007/s41347-026-00678-4)
Supplement: Supplementary file 1 [file 41347_2026_678_MOESM1_ESM.docx]

**Title:** Exploring barriers to crisis support: Considerations for the design of automated digital safety planning interventions.

**Table S1. Mental Health Co-morbidity according to DSM-5 Cross Cutting Symptom Inventory**

|  | **Domain** | **N_Screen_Positive** | **Percent** |
| --- | --- | --- | --- |
| 1 | Depression | 29 | 96.7 |
| 2 | Anger | 24 | 80.0 |
| 3 | Mania | 0 | 0.0 |
| 4 | Anxiety | 30 | 100.0 |
| 5 | Somatic Symptoms | 25 | 83.3 |
| 6 | Suicidal Ideation | 30 | 100.0 |
| 7 | Sleep Problems | 22 | 73.3 |
| 8 | Memory | 15 | 50.0 |
| 9 | Repetitive Thoughts and Behaviors | 28 | 93.3 |
| 10 | Dissociation | 21 | 70.0 |
| 11 | Personality Functioning | 27 | 90.0 |
| 12 | Substance Use | 11 | 36.7 |
| 13 | Psychosis | 0 | 0.0 |

**Table S2. Prompts for the asynchronous remote community (ARC) focus group**

| **ARC focus group 1, Session 3 and ARC focus group 2, Session 4.**  **The role of safety plans** | Some people have filled out something called a safety plan, or, as they are sometimes known, a coping plan before (see attached image). Safety plans are sometimes helpful when people are having suicidal thoughts and want to resist engaging in suicidal behaviors.  For those who might be unfamiliar, safety plans are usually created with a therapist or a counselor, but they can also be created on one's own. They usually include (1) personalized lists of warning signs that suicidal thoughts might be building up and intensifying, (2) a short list of activities people can do on their own to cope, (3) a list of people that a person would be comfortable reaching out to for distractions, (4) a list of people that a person could reach out to for help if they were having difficulty keeping themselves from acting on their suicidal thoughts, (5) a list of professional crisis resources that people can reach out to, (6) a list of ways to put time and distance between oneself and any dangerous objects, and (7) a list of reasons the person experiencing suicidal thoughts might want to remain alive.  *If you've created a safety plan before*:  Were there parts of the creation process you liked or disliked? Why?  Are there parts of the safety plan that you would improve to make it more helpful to you?  What would make you more or less likely to use a safety plan?  *If you've never created a safety plan before*:  What do you imagine would be the hardest parts of creating a safety plan? Is there something that could help make that process easier?  Are there parts of a safety plan that would make you more or less likely to use it? |
| --- | --- |
| **ARC focus group 1, Session 4 and ARC focus group 2, Session 3. Safety planning via text message, impressions** | One way to use technology to help people navigate suicidal crises could be to send text messages. We'd like to know more about your feelings about text messaging. Specifically, imagine that you signed up for a program where you receive automated text messages that helped you create and use a safety plan on your phone.  What are your reactions to the idea of creating, editing, or reviewing a private and highly personalized safety plan via text message? What are some of the reasons for this initial impression?  Are there benefits or drawbacks of a safety planning tool that is delivered over text messaging? |
| **ARC focus group 1, Session 5 and ARC focus group 2, Session 4. Automated messages to help with safety planning** | One way text messaging could be applied to safety planning is to help people write down the things that work for them to "turn down the volume" on their suicidal thoughts. For example, someone may watch a Disney movie to cope with their suicidal thoughts, while another might play a video game to distract themselves from their own thoughts. An automated text messaging program might ask questions like, "What sorts of activities can you do to help take your mind off of problems?" The system would then include the person's response as part of their safety plan.  How would you feel about getting automated messages like this while you create your safety plan? Would some messages be more helpful than others in helping you create a safety plan?  What would make you more or less likely to respond to these messages?  Are there other messages you would want to receive, or other ways a text messaging program could ask you for this information? |
| **ARC focus group 2, Session 5. Help seeking** | Automated safety planning is one tool that can be useful for helping people manage their suicidal thoughts or behaviors, but people may be interested in finding support directly from other people. Some may want support from professional mental health providers such as therapists, psychiatrists, or counselors. Others may want informal support from trusted others in their network (like teachers, clergy, coaches, friends, or family).  Have you ever thought about asking for help for your suicidal thoughts or behaviors either from professionals or from your informal support network (or from both)?  What went into your decision about whether or not to reach out to someone for help, and who to reach out to? |
